# Medical News

**Published:** 1854-02

**Authors:** 


### THE MEDICAL EXAMINER.

PHILADELPHIA, FEBRUARY, 1854.

### MEDICAL NEWS.

From a most interesting discourse on the life and works of the late Robert James Graves, M. D. F. R. S., by Wm. Stokes, M. D., published in the London Medical Times and Gazette, we extract the following: "He was, with me, going round the Hospital, when we entered the convalescent ward. He began to expatiate on the healthy appearance of some who had recovered from severe typhus. "This is all the effect of our good feeding," he exclaimed; and lest, when I am gone, you may be at a loss for an epitaph for me, let me give you one, in three words:—

"*He fed Fevers.*"

"I have given you an epitaph dictated by him in a sportive mood. I will now give you another, which he wrote himself in the earlier part of his last illness, and which he repeated before his death, with directions that it should be engraved upon his tomb:—

Robert James Graves,
Son of Richard Graves, Professor of Divinity,
Who, after a protracted and painful disease, died in the love of God,
and in the faith of Jesus Christ."

---

In the same Journal, January 7th, we have an account of another death from chloroform, communicated by Dr. Wustefeldt, of Neustedt. But one drachm was administered, when insensibility took place. The surgeon had scarcely commenced dividing the skin over the tumor (lipoma) when the girl suddenly fell forwards. All efforts to restore her were found unavailing.

---

We have received a new Journal, published in New York, entitled the American Medical Monthly. It is edited by E. H. Parker, M. D., and conducted by the professors of the New York Medical College. With such a corps of assistants, it cannot fail in being a good Journal. We wish it a long life and prosperity.

Dr. J. B. McCaw has become associated with Dr. Otis, as editor of the Virginia Medical and Surgical Journal.

---

Officers of the Philadelphia County Medical Society for 1854.—At a meeting of the Philadelphia County Medical Society, held January 18th, 1854, the following Officers were elected for the present year:

*President.*—Dr. Thomas Forrest Betton.

*Vice Presidents.*—Dr. George W. Norris, Dr. Thomas H. Yardley.

*Recording Secretary.*—Dr. D. Francis Condie.

*Assistant Secretary.*—Dr. Robert P. Thomas.

*Corresponding Secretary.*—Dr. Henry S. Patterson.

*Treasurer.*—Dr. William Byrd Page.

*Censors.*—Dr. Lewis Rodman, Dr. Nathan L. Hatfield, Dr. E. F. Leake, Dr. William H. Klapp, Dr. William Mayburry.

Published by order,
Robert P. Thomas.
*Philadelphia*, 1854. Assistant Secretary.

---

Obituary.—On the 4th of January, Dr. Samuel McClellan, aged 54 years. Dr. McClellan was formerly Professor of Obstetrics in the Jefferson College, and lectured afterward on the same branch in the Pennsylvania College. He was in the enjoyment of an extensive practice, previous to his sickness. He died, much regretted by his numerous friends, both public and professional.

John Esten Cooke, on the 20th of October, in the 70th year of his age. Dr. Cooke succeeded Dr. Drake in the Chair of the Theory and Practice in Transylvania University, and was subsequently connected with the Medical Institute of Louisville. He was a voluminous writer. Beside his treatise on Pathology and Therapeutics, he wrote for the American Medical Recorder, the Transylvania Journal of Medicine, and occasionally on theological subjects.

Drs. Drake and Cooke were fellow students in Philadelphia in 1805.

In Laurens County, Georgia, where he was a successful practitioner, Dr. A. A. Gittenau, after completing the 35th year of his age. He was a native of Ireland, educated a gentleman and a scholar. He emigrated to this country in 1846, and died of consumption on the 6th of December, 1853.

MEDICAL SOCIETY OF THE STATE OF PENNSYLVANIA.—The annual meeting of the Society, for 1854, will be held in the Borough of *Pottsville,* Schuylkill Co., commencing on *Wednesday, May* 31, at 11 o'clock, A. M. Secretaries of County Societies are requested to send certified copies of the credentials of their delegates, to either of the undersigned, before that date.

HENRY S. PATTERSON, M. D.
No 92 Arch Street, Philadelphia.
ISAAC R. WALKER, M. D.
Spread Eagle P. O., Chester Co.

